# Commentary: MicroRNA-31 Reduces Inflammatory Signaling and Promotes Regeneration in Colon Epithelium, and Delivery of Mimics in Microspheres Reduces Colitis in Mice

**DOI:** 10.3389/fimmu.2019.02649

**Published:** 2019-11-26

**Authors:** Pooja Gupta, Ravi Prakash Yadav, Somesh Baranwal

**Affiliations:** ^1^College of Agriculture, Guru Kashi University, Talwandi Sabo, India; ^2^Department of Biochemistry and Microbial Science, School of Basic and Applied Science, Central University of Punjab, Bathinda, India

**Keywords:** non-coding RNA, microspheres, Hippo signaling, Wnt/b-signaling, experimental colitis

We read the article by Tian et al. (1) on deciphering the molecular mechanisms and therapeutic potential of miR-31 mimic in the progression of Inflammatory Bowel Disease (IBD) with great interest. miR-31, located on chromosome 9p21.3, is a well-established locus that harbors tumor suppressors p15 and p16, regulating the mammalian cell cycle. Based on several reports, miR-31 can function as either an oncogene or a tumor suppressor in a context-dependent manner (2). Moreover, mature miR-31-5p and miR-31-3p bind with various mRNA targets and play a functional role in regulating multiple diseases. In colorectal tissue and cells, miR-31 binds with the 3′UTR of ERK5, RAS1, TGF-β, β-catenin, and several other mRNAs to control radio-resistance, cell migration, and cell proliferation *in-vitro* and tumorigenesis and metastasis *in-vivo*. In contrast, miR-31 shows a tumor-suppressive role in gastric cancer, controlling Rho/ROCK and STAT3 signaling (3). Moreover, circulating miR31 expression is increased in serum samples and has served as a prognostic marker for survival in lung cancer (4).

miR-31 is implicated in several inflammation-associated disorders. In IBD patients, the miR-31 expression signature differentiates Crohn's disease, Ulcerative Colitis, microscopic colitis, and pediatric patients with inflammatory bowel disease. Further, miR-31-expression also controls colonic epithelial cell barrier function and CD8 T-cell response to type I IFN during chronic infection. Also, miR-31 expression positively correlates with LGR5-positive cells, driving intestinal stem cell regeneration under stress conditions. Moreover, miR-31-overexpressing mice demonstrate a significantly higher number of Intestinal Stem Cells (ISC), which have been shown to be able to repair irradiation-induced cell damage (5). In summary, miR-31 is a major regulator of Wnt, BMP, and TGF-β signaling, controlling ISC proliferation, intestinal homeostasis, and colorectal cancer progression. Further, miR-31 also regulates keratinocyte differentiation and is overexpressed in Psoriatic patients, regulating cytokine and chemokine production (6). In the preeclampsia condition, the miR-31-5p expression level is increased in serum samples through TNF-α-mediated activation of nuclear factor-κB, which in turn regulates endothelial dysfunction and vascular remodeling through post-transcriptional downregulations of endothelial nitric oxide synthase (eNOS).

Tian et al. have delineated the precise molecular pathway of miR-31-5p activation in the early inflammatory stage. They determined that miR-31-5p had a profound effect on the activation of canonical Wnt targets proteins and the inhibition of Hippo signaling target proteins in inflammatory bowel disease progression and epithelial regeneration in an *in-vivo* mouse model. Quantitative PCR analysis revealed a significant increase in miR-31-5p expression in Crohn's disease (CD) and Ulcerative Colitis (CD) biopsy tissues, which reverted to the level of healthy control after remission. Treatment with Tumor Necrosis Factor (TNF), interleukin 6 (IL6), and DSS-induced colitis enhanced phosphorylation of STAT3 and p65 (NF-κB), which bound with the miR-31 promoter sequence to activate its expression, as determined by luciferase activity and chromatin immunoprecipitation in malignant LOVO cell lines and organoid derived from mouse colon. In conclusion, inflammatory cytokines dynamically modulate miR-31-5p expression in an early inflammatory condition, and DSS induced colitis in mice and NF-κB signaling directly control miR-31-5p expression. Furthermore, the miR-31-5p level shows a positive correlation with a pathological marker for active UC and CD patients.

miRNA knockout mice emerge as an excellent tool for preclinical disease modeling. Examination of germline knockout (KO) and Villin-Cre-mediated colonic epithelial conditional knockdown (cKO) for miR-31 showed no apparent changes in cell proliferation, apoptosis, and total number of goblet cells in the intestine, while DSS-induced colitis treatment showed a shorter colon length, larger spleen, and higher symptoms of clinical disease. Moreover, miR-31 interacts with the 3′UTR of Interleukin 17 receptor A, Interleukin 6 Signal transducer, and Interleukin- 7 receptor subunit alpha mRNA to control early immune response. Concomitantly, the *in-vivo* permeability for miR-31 knockout is significantly reduced after 3 days of DSS treatment. In summary, their results reveal the essential roles of miR-31 in suppressing early inflammatory response after colitis induction.

Transcriptomics analysis of colonic epithelial cells from wild-type and miR-31 null showed significant changes in the sphingolipid metabolism, TGF-β, and canonical Wnt pathway. Further, there was significant inhibition of β-catenin, Cyclin D1, and Axin2 expression in null mice after colitis induction, suggesting direct regulation of canonical Wnt signaling by miRNA-31. Next, they examined Hippo signaling, which is a critical mediator for tissue regeneration, and found that the Yap/p-YAP ratio was significantly reduced in miR-31 knockout, suggesting the suppression of Hippo signaling following miR-31 activation. Moreover, there was significant upregulation of Lats2, Dlg1, and Dlg5 (downstream target gene of Hippo) following DSS colitis induction. Finally, they confirmed a similar signaling pathway in TNBS-induced colitis (a model for Crohn's disease) and a rescuing effect following overexpression of miR-31 in an *in-vivo* mouse model. In conclusion, they showed that miR-31 activates canonical Wnt signaling and inhibits Hippo signaling to control epithelial regeneration following colitis induction.

Several preclinical studies have demonstrated the potential use of RNA targeting therapeutics such as antisense oligo-nucleotides (ASOs), aptamers, and miRNAs mimics in the prevention of DSS- or TNBS-induced colitis in a mouse model. Administration of these chemically modified oligonucleotides through intracolonic, intraperitoneal, and tail vein injection attenuated clinical features of inflammatory bowel disease (7), yet the biggest hurdle is to achieve safe, efficient, and targeted delivery to specific cells with minimal immunogenic reactions. Emerging reports have suggested ligand conjugation as a novel method of targeted delivery of antisense nucleotides to the specific cells and tissue with minimal off-target effect. Peptosomes are a milk protein (α-lactalbumin)-based delivery system that confers slow release of the drug molecule at the desired location with minimal side effects (8). This paper also highlights the proof in principle for encapsulating a peptosome-based miRNA mimic in a novel light-responsive bioinspired TEMPO-oxidized Konjac Glucomannan (OKGM) (9) (named a OKGM-PS MIR-31 microsphere) into the large intestine of a mouse by injection into the anus and testing its functions in both Wildtype and miR-31 knockout mice with colitis induced by DSS. Enema-based treatment with OKGM-PS MIR-31 microspheres not only attenuates DSS-induced colitis in miR-31 null mice but also plays a protective role in Wild-type mice with induced colitis. Furthermore, OKGM-PS MIR-31 microspheres enhance cell proliferation, reduce immune response, and improve DSS-induced epithelial cell integrity in Wild-type mice. It is not clear from their study how the light-controlled activation step was performed before enema treatment. Given its critical roles in epithelial regeneration along with its low immunogenic activity, miR-31 mimic loaded in dual-layered microspheres seems to be an attractive tool for systemic delivery and slow release of the drug to the target tissue.

Overall, this paper delves into both upstream regulator and downstream effector proteins of miR-31, which play a fundamental role in intestinal inflammation and epithelial cell regeneration, and details novel delivery strategies for miRNA mimics for treating both UC and CD in a preclinical model. It will be interesting to see if miR-31 is involved in other types of mucosal inflammation. Given the diverse roles of miR-31, one of the biggest challenges will be to control off-target detrimental effects. Future studies will unveil the potency, efficacy, pharmacodynamic effects, and safety of this novel and straightforward delivery method of therapeutics in human clinical trials and pave the way for treating chronic inflammatory disease.

## Author Contributions

PG and RY contributed to the first draft of the article. SB and PG conceived, planned, and finalized the manuscript.

### Conflict of Interest

The authors declare that the research was conducted in the absence of any commercial or financial relationships that could be construed as a potential conflict of interest.
